# Depression, anxiety, burnout, and substance use disorders among auto workers and COVID-19 as a stressor in Mexico

**DOI:** 10.3389/fpubh.2025.1723282

**Published:** 2026-02-06

**Authors:** Raúl A. Gutiérrez García, Abraham Sánchez Ruiz, Antonio de Jesús Serrano Carrera, Nancy Alejandra Vaca Rico, Mónica Natalia Arteaga Tovar, María Alicia Zavala Berbena, Marco Antonio Escobar Acevedo, María Abigail Paz Pérez, Kalina Isela Martínez Martínez

**Affiliations:** 1 Facultad de Estudios Superiores, Universidad La Salle Bajío, Salamanca, Guanajuato, Mexico; 2 Kromberg & Schubert, Irapuato, Guanajuato, Mexico; 3 Facultad de Ciencias Sociales y Humanidades, Universidad La Salle Bajío, León, Guanajuato, Mexico; 4 Facultad de Ingenierias y Tecnologias, Universidad La Salle Bajío, León, Guanajuato, Mexico; 5 Departamento de Psicología, Universidad Autónoma de Aguascalientes, Aguascalientes, Aguascalientes, Mexico

**Keywords:** anxiety, automotive workers, burnout, COVID-19, depression, Mexico, occupational stress, substance use disorders

## Abstract

**Introduction:**

The COVID-19 pandemic intensified psychosocial risks, exposing vulnerabilities in labor conditions. Findings aim to inform mental health interventions and workplace policies tailored to industrial workers in post-pandemic settings.

**Methods:**

We evaluated the prevalence of occupational stress and associated psychiatric problems, including anxiety, depression, substance use disorders, and burnout among automotive workers, examining COVID-19 as a significant stressor. Participants were workers in the automotive industry (*N* = 1,020) from two manufacturing plants in Guanajuato, Mexico. We assessed four mental health outcomes: depression, anxiety, substance use disorders, and burnout, using validated instruments.

**Results:**

Multinomial logistic regression was used to examine the associations between COVID-19 stressors and mental health symptoms, while adjusting for sociodemographic characteristics. Prevalence rates were 311 (30.5%) for burnout, 299 (29.3%) for anxiety symptoms, 248 (24.3%) for depressive symptoms, and 142 (13.9%) for substance use problems. Experiencing COVID-19 stressors (adjusted odds ratio (AOR): 2.65, 95% CI: 1.26–3.26) and feeling vulnerable to infection (AOR: 3.34, 95% CI: 2.17–4.06) significantly increased the odds of having comorbid anxiety and depressive symptoms, as well as burnout, compared to workers without these stressors. Despite 763 (74.8%) of workers reporting feeling supported by their company to seek psychological help, only 259 (25.3%) had received mental health treatment in the past year.

**Discussions:**

These findings demonstrate that COVID-19-related stressors significantly impacted automotive workers’ mental health. The substantial gap between symptom prevalence and treatment-seeking, despite perceived workplace support, highlights the need for proactive mental health interventions.

## 1 Introduction

Manufacturing workers globally face elevated mental health risks due to unique occupational stressors including shift work, repetitive tasks, production pressures, and physical demands (1, 2). The global automotive industry employs approximately 10 million workers directly and 50 million indirectly (3), with significant regional concentrations: the European Union employs 13.8 million across the automotive value chain (4), while Mexico’s transport equipment sector has grown to 1.3 million workers, representing 20% of national manufacturing employment and 20% of North American vehicle production (5), represents a critical yet understudied population for mental health research (6, 7). Recent evidence from multiple countries demonstrates concerning mental health prevalence rates among automotive workers: Iranian automotive workers showed 22.6% depression, 24% anxiety, and 29.3% stress symptoms, with 66.7% experiencing high occupational stress (6); Malaysian automotive workers reported mild to severe depression (9.9% severe), anxiety (18.7% severe), and stress symptoms (8); Chinese manufacturing workers demonstrated 25%–30% psychological distress (9); while Vietnamese industrial workers reported 22% depression rates (10).

The Job Demands-Resources model (11) and the psychosocial safety climate framework (12–14) provide theoretical grounding for understanding how workplace factors influence mental health outcomes. In automotive settings specifically, the interaction between high job demands, limited autonomy, and safety pressures creates unique stressors affecting psychological well-being (15, 16). Despite growing recognition of these challenges, evidence-based workplace mental health interventions remain limited (17–19). Barriers include stigma, limited workplace support, cost, and service accessibility (20, 21). Understanding these barriers within specific cultural and organizational contexts is essential for developing effective interventions.

The COVID-19 pandemic fundamentally altered occupational mental health landscapes globally (22, 23). Essential workers, including automotive manufacturing employees who continued on-site work, faced compound stressors: infection risk, job insecurity, and work-family conflict (24–26). Studies across multiple sectors document significant mental health impacts, with healthcare workers showing 30%–40% anxiety/depression rates (27, 28) and industrial workers experiencing similar elevations (29). The pandemic’s prolonged nature created sustained psychological pressure, with evidence of cumulative mental health deterioration across successive waves (30, 31).

In Latin America, only 25% of those with mental disorders receive treatment (32). It has been found that more than 20% of people in Mexico have at least one mental disorder, one in four will suffer from it at least once in their lifetime in the following years (33, 34). This number is expected to increase as a result of COVID-19 (35).

Mexico’s automotive industry, the world’s seventh-largest producer, employs over 900,000 workers (36). The Bajío region, particularly Guanajuato state, represents Mexico’s automotive manufacturing hub. This workforce faces unique challenges: existing mental health service gaps (37, 38), regional violence concerns (39), and recent regulatory changes through NOM-035-STPS-2018 mandating psychosocial risk assessment (40). Mexican healthcare workers during COVID-19 showed elevated mental health impacts (41), yet automotive workers’ experiences remain undocumented.

This study addresses critical gaps by: (1) establishing mental health prevalence (depression, anxiety, burnout, substance use) among Mexican automotive workers; (2) examining COVID-19 as a specific occupational stressor using validated instruments; (3) identifying treatment-seeking patterns and barriers; and (4) comparing outcomes between manufacturing sites. Our findings contribute to global understanding of pandemic impacts on essential manufacturing workers while informing evidence-based interventions for this vulnerable population. Results have implications for international occupational health policy and practice, particularly in emerging economies, balancing industrial growth with worker wellbeing.

## 2 Materials and methods

This study investigated the prevalence and correlates of mental health symptoms among automotive manufacturing workers during the COVID-19 pandemic, with particular focus on depression, anxiety, burnout, and substance use patterns. The research employed validated psychometric instruments to assess mental health outcomes and examined the role of pandemic-related stressors as potential risk factors within this essential worker population.

### 2.1 Study design

This cross-sectional study was conducted between July and November 2022 to assess mental health prevalence and associated factors among automotive manufacturing workers in Guanajuato, Mexico. The research protocol adhered to the Declaration of Helsinki ethical principles. The personal information, classified as sensitive by the Federal Law for the Protection of Personal Data in the Possession of Private Parties, was treated confidentially.

### 2.2 Participants and recruitment

The total workforce population of approximately 7,000 employees across both facilities, we calculated a required sample size of 1,020 participants to achieve 95% confidence level with a 2.837% margin of error. Recruitment employed a multi-modal approach combining institutional email invitations and in-person recruitment sessions conducted by human resources and occupational health personnel. Although 1,119 workers were invited, 101 decided not to participate.

The inclusion criteria comprised (1) a minimum age of 18 years, (2) current employment in either manufacturing facility, and (3) voluntary informed consent. The exclusion criteria consisted of temporary contractors and individuals on medical or administrative leave during the data collection period. The final analytical sample consisted of 1,020 participants, representing a response rate of 91.1% of the target sample.

### 2.3 Data collection procedures

Data collection utilized a self-administered online survey deployed through QuestionPro® platform. The survey incorporated adaptive questioning logic to minimize completion time while maintaining comprehensive assessment coverage. Prior to full deployment, the instrument underwent pilot testing with 30 workers to assess comprehension, technical functionality, and completion time.

Participants received unique, anonymized access codes ensuring confidentiality while preventing duplicate responses. The survey remained accessible 24/7 during the collection period to accommodate varying work shifts. Automated reminder emails were sent at 7 and 14 days to non-responders. All responses were encrypted and stored on secure servers compliant with institutional data protection protocols.

### 2.4 Measures

The comprehensive assessment battery included internationally validated instruments adapted and validated for Mexican Spanish-speaking populations:

#### 2.4.1 Depression assessment

The Patient Health Questionnaire-9 (PHQ-9) measured depressive symptom severity over the preceding 2 weeks (42). Items are scored 0–3, yielding total scores ranging 0–27, with established cutoffs: minimal (0–4), mild (5–9), moderate (10–14), moderately severe (15–19), and severe (20–27) depression. Internal consistency in this sample was excellent (Cronbach’s *α* = 0.83).

#### 2.4.2 Anxiety assessment

The Generalized Anxiety Disorder-7 (GAD-7) scale evaluated anxiety symptoms, including nervousness, restlessness, and catastrophic thinking (43). The instrument demonstrated high internal consistency (α = 0.92) with validated cutoffs: minimal (0–4), mild (5–9), moderate (10–14), and severe (≥15) anxiety.

#### 2.4.3 Burnout syndrome

The Maslach Burnout Inventory (MBI) assessed three dimensions of occupational burnout (44): emotional exhaustion (9 items, *α* = 0.837), depersonalization (5 items, *α* = 0.869), and personal accomplishment (8 items, *α* = 0.881). Dimensional scores were calculated following standardized protocols with normative comparisons for manufacturing workers. Burnout was defined as high emotional exhaustion (≥27), high depersonalization (≥10), and low personal accomplishment (≤33).

#### 2.4.4 Substance use screening

The Alcohol, Smoking and Substance Involvement Screening Test (ASSIST) evaluated lifetime and past three-month use patterns for 10 substance categories (45). Risk scores were categorized as low (0–3), moderate (4–26), or high (≥27) for alcohol, and adjusted thresholds for other substances per WHO guidelines.

#### 2.4.5 COVID-19 stress impact

The Impact of Event Scale-Revised (IES-R) measured pandemic-related traumatic stress across three domains: intrusion (8 items), avoidance (8 items), and hyperarousal (6 items) (46). Items were rated 0–4 regarding distress intensity during the previous week, with subscale scores indicating subclinical (<33) or probable PTSD (≥33) symptomatology.

#### 2.4.6 Sociodemographic and occupational variables

Structured questions assessed age, gender, education, marital status, job position (production line, technical, administrative), shift schedule, tenure, and COVID-19 exposure history, including personal infection, family losses, and vaccination status.

### 2.5 Statistical analysis

Data analysis employed SPSS version 25.0 (IBM Corp., Armonk, NY). Descriptive statistics characterized the sample demographics and prevalence rates with 95% confidence intervals. Internal consistency was verified using Cronbach’s alpha, with values ≥0.70 considered acceptable.

Bivariate analyses examined associations between mental health outcomes and potential risk factors using Chi-square tests for categorical variables and *Student’s t-test* for continuous variables. The *Kolmogorov–Smirnov* normality test was performed for all quantitative variables included in the analyses. In all cases, the *p*-values were greater than 0.05, indicating that the null hypothesis of normality is not rejected. Multinomial logistic regression was used to analyze the associations between COVID-19 stressors and different profiles of psychological distress. The reference category was the absence of clinically significant symptoms. The final model included the following covariates: age, sex, educational level, presence of medical comorbidities, exposure to COVID-19-related stressors, and perception of vulnerability, adjusting for sociodemographic covariates identified as significant (*p* < 0.10) in bivariate analyses.

Model assumptions were verified including multicollinearity assessment (VIF < 5), and goodness-of-fit evaluated using Hosmer-Lemeshow tests. Adjusted odds ratios (AOR) with 95% confidence intervals were calculated. Statistical significance was set at *p* < 0.05 for all analyses. Missing data patterns were examined, with complete case analysis employed given <5% missingness across variables.

## 3 Results

### 3.1 Sample characteristics

The final sample comprised 1,020 automotive manufacturing workers from two plants in Guanajuato, Mexico: Irapuato (*n* = 607, 59.5%) and San Francisco del Rincón (*n* = 413, 40.5%). Participants had a mean age of 30.4 years (SD = 10.1), with 58.1% identifying as male (*n* = 593) and 41.9% as female (*n* = 427). The majority (51.0%, *n* = 520) were married or in domestic partnerships, 39.0% (*n* = 398) were single, and 10.0% (*n* = 102) were separated, divorced, or widowed. Educational attainment varied, with 39.0% (*n* = 398) completing high school, 33.0% (*n* = 337) technical training, and 17.0% (*n* = 173) holding undergraduate or postgraduate degrees. Over half (57.0%, *n* = 581) reported having children (mean = 2.8 children). Regarding occupational characteristics, 56.0% (*n* = 571) worked as direct production workers, 32.0% (*n* = 326) in indirect/operational support roles, and 12.0% (*n* = 122) in administrative positions. The production department employed 77.0% (*n* = 785) of participants, with the remaining workers distributed across quality control, human resources, logistics, and other departments. Half worked morning shifts (50.0%, *n* = 510), 42.0% (*n* = 428) afternoon shifts, and 8.0% (*n* = 82) rotating schedules. Complete demographic and baseline mental health scores are presented in Table 1.

**Table 1:** Sociodemographic characteristics and mental health indicators of automotive sector workers (*N* = 1,020).

Characteristic	*n* (%) or Mean (SD)
Age (years)	30.4 (1.01)
Age groups	
18–24	306 (30.1)
25–34	357 (35.9)
35–44	204 (20.3)
45+	153 (15.6)
Gender	
Male	593 (58.1)
Female	427 (41.9)
Sexual orientation	
Heterosexual	977 (95.7)
Other	43 (4.3)
Marital status	
Married/Cohabitating	113 (51.1)
Single	285 (39.1)
Separated/Divorced/Widowed	102 (9.8)
Has children	
Yes	581 (57.4)
No	439 (43.6)
Education level	
High school or less	398 (39.8)
Technical training	337 (33.9)
Bachelor’s degree or higher	173 (17.9)
Mental health indicators	Mean (SD)
Depression (PHQ-9)	24.27 (6.34)
Anxiety (GAD-7)	29.31 (4.23)
Burnout (MBI)	30.53 (5.56)
Substance use (ASSIST)	19.93 (7.28)
COVID-19 stress (IES-R)	34.19 (6.11)

### 3.2 Mental health prevalence

Mental health symptoms were prevalent across the sample, with 34.2% experiencing COVID-19–related traumatic stress symptoms were prevalent across the sample, with 34.2% (*n* = 349) experiencing clinically significant symptoms based on IES-R scores ≥33. Burnout affected 30.5% (*n* = 311) of workers, with emotional exhaustion being the predominant dimension. Anxiety symptoms (GAD-7 ≥ 10) were present in 29.3% (*n* = 299) of participants, while 24.3% (*n* = 248) met criteria for moderate to severe depression (PHQ-9 ≥ 10). Substance use problems, assessed via ASSIST, were identified in 13.9% (*n* = 142) of workers, primarily involving tobacco (38.0% lifetime use, *n* = 388) and alcohol (73.0% lifetime use, *n* = 745).

### 3.3 Between-plant comparisons

Significant differences emerged between manufacturing sites Student’s *t*-test (Table 2). Workers from San Francisco del Rincón reported a higher prevalence of COVID-19–related traumatic stress symptoms (37.7%, *n* = 156) compared to those from Irapuato (31.7%, *n* = 192) (*p* = 0.012). Similarly, work-related burnout was more common in San Francisco (33.1%, *n* = 137) than in Irapuato (27.8%, *n* = 169) (*p* < 0.001). Anxiety prevalence was comparable between sites, with 30.1% (*n* = 124) in San Francisco and 28.4% (*n* = 172) in Irapuato (*p* < 0.001). In contrast, depression symptoms showed a slightly higher prevalence in Irapuato (25.1%, *n* = 152) compared to San Francisco (23.5%, *n* = 97) (*p* < 0.001).

**Table 2:** Prevalence of mental health symptoms by manufacturing plant location with Student’s *t*-test and Chi-square.

Variable	Total (%)	San Francisco (%)	Irapuato (%)	*p*-value
Work-related traumatic stress (COVID-19 event)	34.2	37.7	31.7	0.012
Work-related stress symptoms (MBI)	30.5	33.1	27.8	<0.001
Anxiety symptoms (GAD-7)	29.3	30.1	28.4	<0.001
Depressive symptoms (PHQ-9)	24.3	23.5	25.1	<0.001

### 3.4 Risk factors and help-seeking patterns

Lifetime violence exposure was reported by 62.7% (*n* = 640) of participants, with higher rates in San Francisco del Rincón (66.0%, *n* = 273) compared to Irapuato (62.4%, *n* = 379) (*p* < 0.001; Table 3). Despite high symptom prevalence, only 25.3% (*n* = 258) had accessed mental health services in the past year, with Irapuato workers showing higher utilization (27.7%, *n* = 168) compared to San Francisco (22.9%, *n* = 95) (*p* < 0.001). Notably, 74.8% (*n* = 763) felt their employer would support help-seeking, though this perception was stronger among workers in Irapuato (77.3%, *n* = 469) than in San Francisco (72.2%, *n* = 298) (*p* = 0.004), with Student’s *t*-test.

**Table 3:** Substance use, violence exposure, and help-seeking behaviors by manufacturing plant location with Student’s *t*-test and Chi-square.

Variable	Total (%)	San Francisco (%)	Irapuato (%)	*p*-value
Substance use problems (ASSIST)	13.9	15.1	12.8	<0.001
Exposure to violence (lifetime)	62.7	66.0	62.4	<0.001
Received mental health treatment (past 12 months)	25.3	22.9	27.7	<0.001
Feels supported by company to seek help	74.8	72.2	77.3	0.004

### 3.5 COVID-19 stressor associations

**Figure 1:**
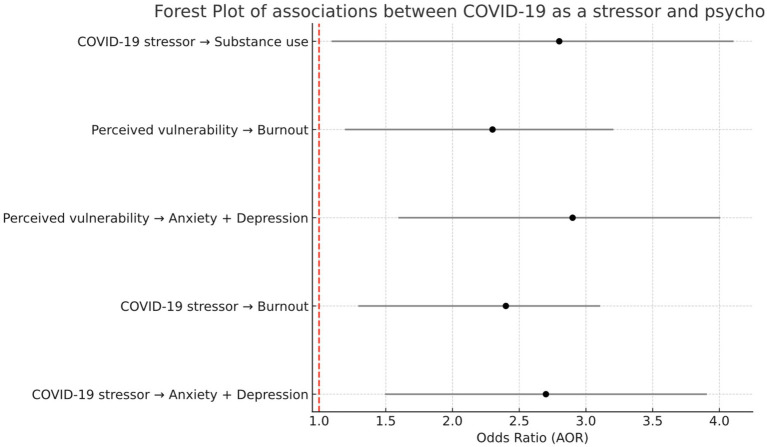
Forest plot of adjusted odds ratio (AOR) and 95% confidence intervals associated with COVID-19 stressors among automotive workers.

Multinomial logistic regression revealed that COVID-19 stressors significantly increased odds of comorbid mental health symptoms (Figure 1). Workers experiencing pandemic-related stressors had 2.65 times higher odds (95% CI: 1.26–3.26) of concurrent anxiety and depression compared to those without such stressors. Feeling vulnerable to COVID-19 infection showed the strongest association, with 3.34 times higher odds (95% CI: 2.17–4.06) of multiple mental health symptoms after adjusting for sociodemographic factors.

### 3.6 Barriers to mental health services

Primary barriers to seeking professional help included wanting to handle problems independently (mean importance = 1.98), preference for informal support (2.18), cost concerns (2.25), administrative obstacles (2.11), sociocultural stigma (2.81), and fear of job repercussions (2.61). These barriers persisted despite three-quarters of workers perceiving organizational support for mental health help-seeking.

## 4 Discussion

This study documents a substantial mental health burden among Mexican automotive workers during the COVID-19 pandemic, with prevalence rates of 34.2% for work-related stress (*n* = 349), 30.5% for burnout (*n* = 311), 29.3% for anxiety (*n* = 299), and 24.3% for depression (*n* = 248).

These findings contribute to the limited but growing body of research examining pandemic impacts on industrial workers who maintained essential production while facing unique occupational and psychosocial stressors, warranting urgent attention from both occupational health and public health perspectives.

Significant differences emerged between manufacturing sites despite shared corporate policies and regional location. San Francisco workers showed higher COVID-19-related stress (37.7% vs. 31.7%, *p* = 0.012) and burnout (33.1% vs. 27.8%, *p* < 0.001) compared to Irapuato, yet paradoxically reported lower mental health service utilization (22.9% vs. 27.7%, *p* < 0.001) and perceived organizational support (72.2% vs. 77.3%, *p* = 0.004). These variations suggest local workplace cultures, community factors, or workforce composition significantly influence both mental health outcomes and help-seeking behaviors, highlighting that one-size-fits-all interventions may be insufficient even within the same company.

Perhaps our most concerning finding is the disconnect between perceived support and actual help-seeking behavior. While 74.8% of workers reported feeling supported by their company to seek psychological help, only 25.3% had accessed mental health services in the past year, despite approximately one-third reporting clinically significant symptoms. The primary barriers identified, desire for self-reliance (1.98), preference for informal support (2.18), and fear of job repercussions (2.61), reflect deeply embedded cultural and structural factors that organizational support alone cannot overcome.

### 4.1 Principal findings and international comparisons

Our depression prevalence (24.3%) closely parallels findings from U.S. automotive workers [22.3%; (29)] but falls below rates reported among U.S. essential workers more broadly, where 74%–78% reported anxiety or depression symptoms (47). The anxiety prevalence in our sample (29.3%) exceeds that reported in U.S. automotive workers [23.6%; (29)] but is substantially lower than the combined anxiety/depression rates in Brazilian and Spanish essential workers, where 27.4% experienced both conditions and 11.6% anxiety alone (24). Notably, De Boni et al. found that Brazilian essential workers had significantly higher odds of depression (AOR 2.89), anxiety (AOR 2.81), and comorbidity (AOR 5.99) compared to Spanish workers, suggesting that socioeconomic inequalities in middle-income countries may exacerbate pandemic mental health impacts.

The burnout rate of 30.5% in our sample significantly exceeds that reported among New Zealand essential workers post-lockdown [14%, (48)], though direct comparison is complicated by different measurement approaches and timing. Indian automotive workers also experienced substantial psychosocial impacts during COVID-19, with job anxiety emerging as a three-dimensional construct encompassing stimuli-related, socially-related, and health-related stressors (49). Finnish workers showed fluctuating COVID-19 anxiety across pandemic waves, with the highest levels during lockdown periods (50), highlighting the temporal dynamics of pandemic-related distress.

The global framework synthesis by Sheridan Rains et al. (51), analyzing data from 28 countries, documented widespread deterioration in mental health symptoms and service access challenges during early pandemic phases, consistent with our findings. The International Labour Office (52) further confirmed differential pandemic impacts across worker categories and regions, with essential workers in developing economies facing compounded vulnerabilities.

### 4.2 COVID-19 as an occupational stressor

Our finding that COVID-19 stressors increased odds of comorbid anxiety and depression by 2.65-fold (95% CI: 1.26–3.26) aligns with international evidence on pandemic mental health impacts among essential workers. De Boni et al. (24) demonstrated that an unhealthy lifestyle during confinement dramatically increased odds of depression (AOR 4.00), anxiety (AOR 2.39), and comorbidity (AOR 8.30), suggesting that pandemic-related behavioral changes mediate mental health outcomes. Similarly, Naghavi-Konjin et al. (53) found complex relationships between COVID-19 risk perception and anxiety among Iranian manufacturing workers, where higher risk awareness paradoxically reduced anxiety, possibly through enhanced control perceptions.

The stronger association with feeling vulnerable (AOR: 3.34, 95% CI: 2.17–4.06) in our study parallels findings across essential worker populations. Amsalem et al. (47) identified vulnerability factors, including younger age, female gender, and transgender identity, as predictors of higher symptom severity among U.S. essential workers. This vulnerability-distress relationship was particularly pronounced among healthcare workers globally, with Mexican healthcare workers showing similar patterns (41), suggesting universal psychological mechanisms operating across essential worker categories.

Our findings must be understood within the broader context of pandemic impacts on developing economies. The ILO (52) documented how workers in developing countries faced pronounced income losses with minimal stimulus support, creating a “vicious circle” affecting well-being and recovery. This is particularly relevant for Mexican automotive workers who, despite maintaining employment, operated within systems characterized by “decent work deficits”: inadequate income or economic insecurity, excessive working hours or unstable schedules, lack of occupational safety and health protections, limited access to social protection or health services, absence of voice, representation, or workplace participation, exposure to discrimination, violence, or harassment, and constraints on personal development or work life balance; and limited social protection factors that likely amplified the psychological burden of pandemic stressors beyond what objective exposure alone would predict.

The psychosocial pathways linking COVID-19 exposure to mental health outcomes involve multiple mechanisms. Building on Oksanen et al.’s (50) identification of psychological distress, work exhaustion, and technostress as predictors, our study demonstrates that perceived vulnerability operates independently from actual exposure, with subjective threat appraisal (AOR: 3.34) showing stronger associations than objective stressor exposure (AOR: 2.65). This suggests interventions targeting threat perception and coping resources may be as important as reducing actual exposure risks.

### 4.3 Limitations

While this study provides valuable insights, several limitations merit consideration. The cross-sectional design prevents causal inference about COVID-19 stressors and mental health outcomes. Self-report measures in a workplace setting may underestimate true prevalence due to stigma concerns. Our sample from two plants in Guanajuato limits generalizability, and the absence of pre-pandemic baseline data prevents determining whether observed rates reflect COVID-specific increases or ongoing occupational challenges. Additionally, unmeasured factors including work conditions, home stressors, and mental health history may confound observed relationships. Despite these constraints, our findings document substantial mental health burden requiring urgent intervention in Mexican automotive workers. We acknowledge that unmeasured contextual factors such as differences in sociodemographic characteristics between worksites, variability in job demands, pre-existing mental health conditions, and unequal access to support services may act as confounders in the observed associations. Additionally, methodological constraints inherent to the study design, including its cross-sectional nature, reliance on self-reported data, and lack of longitudinal follow-up, limit causal inference.

## 5 Conclusion

This study documented the prevalence of mental health symptoms among Mexican automotive workers during COVID-19, with approximately one-third experiencing work-related stress and burnout, one-quarter reporting clinically significant anxiety and depression, and 14% engaging in hazardous substance use. The association between COVID-19 stressors and increased mental health symptoms (AOR: 2.65) underscores the pandemic’s amplification of existing occupational vulnerabilities in manufacturing settings. These findings highlight the urgent need for culturally-adapted workplace mental health interventions in Mexico’s automotive sector, including enhanced psychosocial safety protocols, accessible mental health services tailored for shift workers, and organizational strategies to reduce stigma. Specifically, service use includes individual psychotherapy, and mental health counseling or brief psychological support, and, in some cases, employer-provided wellness or stress-management programs. Implementation of evidence-based workplace interventions could substantially reduce psychological morbidity while improving productivity and retention in this essential industrial workforce.

On-site psychological services, available during all shifts (morning, afternoon, and night), with a focus on immediate care, accessibility, and confidentiality. Extended coverage responds to the need to provide support to students, teachers, or workers who, due to their schedules, cannot access traditional services. Peer support programs based on psychosocial intervention models that train students or volunteer collaborators as initial emotional support agents. These programs promote a sense of community, reduce stigma, and facilitate the timely detection of people at risk. Psychoeducation and mental health promotion campaigns aimed at informing people about common symptoms, healthy coping mechanisms, and available care options. These campaigns should be culturally relevant and use digital, print, and in-person media to ensure their reach. Effective integration of mental health services with occupational health units, both in educational institutions and workplaces. This coordination allows for continuous monitoring of emotional well-being, facilitates early interventions, and promotes healthy work and study environments.

## Data Availability

The raw data supporting the conclusions of this article will be made available by the authors, without undue reservation. The study team can provide de-identified raw data upon request, subject to compliance with participant confidentiality and institutional ethics approval conditions.

## References

[1] Brown JP Martin D Nagaria Z Verceles AC Jobe SL Wickwire EM. Mental health consequences of shift work: an updated review. Curr Psychiatry Rep. (2020) 22:7. doi: 10.1007/s11920-020-1131-z, 31955278

[2] Roche AM Pidd K Fischer JA Lee N Scarfe A Kostadinov V. Men, work, and mental health: a systematic review of depression in male-dominated industries and occupations. Saf Health Work. (2016) 7:268–83. doi: 10.1016/j.shaw.2016.04.005, 27924229 PMC5127922

[3] Willums J-O Midttun A Staurem E. The automotive industry: meandering towards green transition in the European Union and the United States In: Midttun A Witoszek N, editors. Energy and transport in green transition. London: Routledge (2015)

[4] Helmold M. New work in the automotive industry In: Helmold M, editor. New work, transformational and virtual leadership. Singapore: Springer International Publishing (2021). 157–69.

[5] Sanginés J. C. Russo M. Simonazzi A. 2021. Mexico’s automotive industry: a success story? (SSRN Scholarly Paper No. 3952148). Social Science Research Network. Available online at: https://papers.ssrn.com/abstract=3952148 (Accessed January 9, 2026).

[6] Eftekhari S Karimi L Sharifian SA Aminian O Rasooli SR Zia G. Association between occupational stress and mental health in an automobile manufacturing factory in Iran. Int J Occup Saf Health. (2025) 15:1–14. doi: 10.3126/ijosh.v15i1.61572

[7] Omair M Ullah M Ganguly B Noor S Maqsood S Sarkar B. The quantitative analysis of workers’ stress due to working environment in the production system of the automobile part manufacturing industry. Mathematics. (2019) 7:627. doi: 10.3390/math7070627

[8] Abdul Ghapa NA Hashim H Rasdi I Zainal Abidin E. The relationship between safety culture maturity and mental health among workers in automotive industry. Malays J Med Health Sci. (2023) 19:101–8. doi: 10.47836/mjmhs.19.s14.11

[9] Meng J Meng X. Psychological distress among Chinese manufacturing employees: prevalence and a symptom network analysis. PsyCh Journal. (2025) 14:603–13. doi: 10.1002/pchj.70015, 40300878 PMC12318592

[10] Do HN Nguyen AT Nguyen HQT Bui TP Nguyen QV Tran NTT . Depressive symptoms, suicidal ideation, and mental health service use of industrial workers: evidence from Vietnam. Int J Environ Res Public Health. (2020) 17:2929. doi: 10.3390/ijerph17082929, 32340335 PMC7216084

[11] Bakker AB Demerouti E. Job demands–resources theory: taking stock and looking forward. J Occup Health Psychol. (2017) 22:273–85. doi: 10.1037/ocp0000056, 27732008

[12] Dollard MF Bakker AB. Psychosocial safety climate as a precursor to conducive work environments, psychological health problems, and employee engagement. J Occup Organ Psychol. (2010) 83:579–99. doi: 10.1348/096317909X470690

[13] Hall GB Dollard MF Coward J. Psychosocial safety climate: development of the PSC-12. Int J Stress Manag. (2010) 17:353–83. doi: 10.1037/a0021320

[14] Law R Dollard MF Tuckey MR Dormann C. Psychosocial safety climate as a lead indicator of workplace bullying and harassment, job resources, psychological health and employee engagement. Accid Anal Prev. (2011) 43:1782–93. doi: 10.1016/j.aap.2011.04.010 21658506

[15] Chenarboo FJ Hekmatshoar R Fallahi M. The influence of physical and mental workload on the safe behavior of employees in the automobile industry. Heliyon. (2022) 8:e11034. doi: 10.1016/j.heliyon.2022.e11034, 36276745 PMC9582718

[16] Mollaei N Fujao C Silva L Rodrigues J Cepeda C Gamboa H. Human-centered explainable artificial intelligence: automotive occupational health protection profiles in prevention musculoskeletal symptoms. Int J Environ Res Public Health. (2022) 19:9552. doi: 10.3390/ijerph19159552, 35954919 PMC9368597

[17] Hulls P. M. Richmond R. C. Martin R. M. Chavez-Ugalde Y. Vocht F. De. (2022). Workplace interventions that aim to improve employee health and well-being in male-dominated industries: a systematic review. Occup Environ Med, 79, 77–87. doi:10.1136/oemed-2020-107314, 34035181 PMC8785069

[18] Joyce S Modini M Christensen H Mykletun A Bryant R Mitchell PB . Workplace interventions for common mental disorders: a systematic meta-review. Psychol Med. (2016) 46:683–97. doi: 10.1017/S0033291715002408, 26620157

[19] LaMontagne AD Martin A Page KM Reavley NJ Noblet AJ Milner AJ . Workplace mental health: developing an integrated intervention approach. BMC Psychiatry. (2014) 14:131. doi: 10.1186/1471-244X-14-131, 24884425 PMC4024273

[20] Kristman VL Lowey J Fraser L Armstrong S Sawula S. A multi-faceted community intervention is associated with knowledge and standards of workplace mental health: the superior mental wellness @ work study. BMC Public Health. (2019) 19:638. doi: 10.1186/s12889-019-6976-x, 31126273 PMC6534893

[21] Paterson C Leduc C Maxwell M Aust B Strachan H O’Connor A . Barriers and facilitators to implementing workplace interventions to promote mental health: qualitative evidence synthesis. Syst Rev. (2024) 13:152. doi: 10.1186/s13643-024-02569-2, 38849924 PMC11157821

[22] Penninx BWJH Benros ME Klein RS Vinkers CH. How COVID-19 shaped mental health: from infection to pandemic effects. Nat Med. (2022) 28:2027–37. doi: 10.1038/s41591-022-02028-2, 36192553 PMC9711928

[23] WHO. Mental health and COVID-19: early evidence of the pandemic’s impact: scientific brief. Geneva: World Health Organization (2022).

[24] de Boni RB Balanzá-Martínez V Mota JC de Azevedo Cardoso T Ballester P Atienza-Carbonell B . Depression, anxiety, and lifestyle among essential workers: a web survey from Brazil and Spain during the COVID-19 pandemic. J Med Internet Res. (2020) 22:e22835. doi: 10.2196/22835, 33038075 PMC7641648

[25] Giorgi G Lecca LI Alessio F Finstad GL Bondanini G Lulli LG . COVID-19-related mental health effects in the workplace: a narrative review. Int J Environ Res Public Health. (2020) 17:7857. doi: 10.3390/ijerph17217857, 33120930 PMC7663773

[26] Manchia M Gathier AW Yapici-Eser H Schmidt MV De Quervain D Van Amelsvoort T . The impact of the prolonged COVID-19 pandemic on stress resilience and mental health: a critical review across waves. Eur Neuropsychopharmacol. (2022) 55:22–83. doi: 10.1016/j.euroneuro.2021.10.864, 34818601 PMC8554139

[27] Eyles E Moran P Okolie C Dekel D Macleod-Hall C Webb RT . Systematic review of the impact of the COVID-19 pandemic on suicidal behaviour amongst health and social care workers across the world. J Affect Disord Rep. (2021) 6:100271. doi: 10.1016/j.jadr.2021.100271, 34841385 PMC8607051

[28] Shechter A Diaz F Moise N Anstey DE Ye S Agarwal S . Psychological distress, coping behaviors, and preferences for support among New York healthcare workers during the COVID-19 pandemic. Gen Hosp Psychiatry. (2020) 66:1–8. doi: 10.1016/j.genhosppsych.2020.06.007, 32590254 PMC7297159

[29] Laskaris Z Fleischer NL Burgard S Eisenberg JN. Personal and work-related factors associated with mental health among auto workers during the COVID-19 pandemic in the United States. Prev Med Rep. (2022) 30:102001. doi: 10.1016/j.pmedr.2022.102001, 36189126 PMC9512522

[30] Prati G Mancini AD. The psychological impact of COVID-19 pandemic lockdowns: a review and meta-analysis of longitudinal studies and natural experiments. Psychol Med. (2021) 51:201–11. doi: 10.1017/S0033291721000015, 33436130 PMC7844215

[31] Vindegaard N Benros ME. COVID-19 pandemic and mental health consequences: systematic review of the current evidence. Brain Behav Immun. (2020) 89:531–42. doi: 10.1016/j.bbi.2020.05.048, 32485289 PMC7260522

[32] Kohn R Ali AA Puac-Polanco V Figueroa C López-Soto V Morgan K . Mental health in the Americas: an overview of the treatment gap. Rev Panam Salud Publica. (2018) 42:e165. doi: 10.26633/RPSP.2018.165, 31093193 PMC6386160

[33] Medina-Mora ME Borges G Munoz CL Benjet C Jaimes JB Bautista CF . Prevalencia de trastornos mentales y uso de servicios: Resultados de la Encuesta Nacional de Epidemiología Psiquiátrica en México. Salud Mental. (2003) 26:1–16.

[34] Orozco R Borges G Medina-Mora ME Aguilar-Gaxiola S Breslau J. A cross-National Study on prevalence of mental disorders, service use, and adequacy of treatment among Mexican and Mexican American populations. Am J Public Health. (2013) 103:1610–8. doi: 10.2105/AJPH.2012.301169, 23865664 PMC3780667

[35] Moreno C Wykes T Galderisi S Nordentoft M Crossley N Jones N . How mental health care should change as a consequence of the COVID-19 pandemic. Lancet Psychiatry. (2020) 7:813–24. doi: 10.1016/S2215-0366(20)30307-2, 32682460 PMC7365642

[36] Guzman-Anaya L. The role of Japanese cooperation in the transition to electric vehicle production in Mexico In: Guzman-Anaya L, editor. Japanese cooperation and supporting industry in Mexico’s automotive sector: USMCA, Covid-19 disruptions, and electric vehicle production. Singapore: Springer Nature (2023). 31–52.

[37] Benjet C Borges G Méndez E Albor Y Casanova L Orozco R . Eight-year incidence of psychiatric disorders and service use from adolescence to early adulthood: longitudinal follow-up of the Mexican adolescent mental health survey. Eur Child Adolesc Psychiatry. (2016) 25:163–73. doi: 10.1007/s00787-015-0721-5, 26009150

[38] Medina-Mora ME Borges G Benjet C Lara MDC Rojas E Fleiz C . Estudio de los trastornos mentales en México: Resultados de la Encuesta Mundial de Salud Mental. Epidemiología de Los Trastornos Mentales En América Latina y El Caribe. (2009):79–89.

[39] Reyes Guzmán G Escobar Acevedo MA Rostro Hernández PE. Mexico: remittances, organized crime and US drug overdose crisis in borderlands (2015-2021). Norteamérica. (2022) 18. doi: 10.22201/cisan.24487228e.2023.1.602

[40] STPS. 2018. Norma Oficial Mexicana NOM-035-STPS-2018, Factores de riesgo psicosocial en el trabajo-Identificación, análisis y prevención. Diario Oficial de La Federación. Available online at: https://asinom.stps.gob.mx/upload/nom/48.pdf (Accessed January 9, 2026).

[41] Robles R Morales-Chainé S Bosch A Astudillo-García C Feria M Infante S . Mental health problems among COVID-19 frontline healthcare workers and the other country-level epidemics: the case of Mexico. Int J Environ Res Public Health. (2022) 19:421. doi: 10.3390/ijerph19010421, 35010679 PMC8744587

[42] Kroenke K Spitzer RL Williams JBW. The PHQ-9: validity of a brief depression severity measure. J Gen Intern Med. (2001) 16:606–13. doi: 10.1046/j.1525-1497.2001.016009606.x, 11556941 PMC1495268

[43] Spitzer RL Kroenke K Williams JBW Löwe B. A brief measure for assessing generalized anxiety disorder: the GAD-7. Arch Intern Med. (2006) 166:1092. doi: 10.1001/archinte.166.10.1092, 16717171

[44] Maslach C Jackson SE Leiter MP. Maslach burnout inventory: third edition In: Zalaquett CP Wood RJ, editors. Evaluating stress: a book of resources. Lanham, MD: Scarecrow Education (1997). 191–218.

[45] Who Assist Working Group. The alcohol, smoking and substance involvement screening test (ASSIST): development, reliability and feasibility. Addiction. (2002) 97:1183–94. doi: 10.1046/j.1360-0443.2002.00185.x 12199834

[46] Weiss DS. The impact of event scale: revised In: Wilson JP Tang CS, editors. Cross-cultural assessment of psychological trauma and PTSD. New York, NY: Springer US (2007). 219–38.

[47] Amsalem D Fisch CT Wall M Choi CJ Lazarov A Markowitz JC . Anxiety and depression symptoms among young U.S. essential workers during the COVID-19 pandemic. Psychiatr Serv. (2023) 74:1010–8. doi: 10.1176/appi.ps.20220530, 37042105

[48] Haar J O’Kane C. A post-lockdown study of burnout risk amongst New Zealand essential workers. Soc Sci Med. (2022) 306:115157–9. doi: 10.1016/j.socscimed.2022.115157, 35738197

[49] Swain D John T Jena LK Gaan N Jena A. Influence of job anxiety among the blue-collar technical workforce of Indian manufacturing industries: a post-COVID perspective In: Mishra P Sharma A Khanra S Kundu SK Mishra SK, editors. Digital economy post COVID-19 era. Singapore: Springer Nature (2023). 695–718. doi: 10.1007/978-981-99-0197-5_44

[50] Oksanen A Oksa R Celuch M Cvetkovic A Savolainen I. COVID-19 anxiety and wellbeing at work in Finland during 2020-2022: a 5-wave longitudinal survey study. Int J Environ Res Public Health. (2022) 20:680. doi: 10.3390/ijerph20010680, 36612998 PMC9819787

[51] Sheridan Rains L Johnson S Barnett P Steare T Needle JJ Carr S . Early impacts of the COVID-19 pandemic on mental health care and on people with mental health conditions: framework synthesis of international experiences and responses. Soc Psychiatry Psychiatr Epidemiol. (2021) 56:13–24. doi: 10.1007/s00127-020-01924-7, 32804258 PMC7429938

[52] International Labour Office. World employment and social outlook: trends 2022. Geneva: International Labour Organization (2022).

[53] Naghavi-Konjin Z Keshavarz V Koushki KG Niko AY Charati JY Gorgani Firouzjaei M. Survey of the correlation between COVID-19 risk perception and anxiety among manufacturing workers. J Health Safety Work. (2024) 14:54–71. doi: 10.18502/jhsw.v14i1.17121

